# Proposing Multiregional Diagnostic Reference Levels for Common CT Angiography Examinations in Saudi Arabia

**DOI:** 10.3390/diagnostics14141523

**Published:** 2024-07-15

**Authors:** Ali Alhailiy, Essam Alkhybari, Mohammed Alshuhri, Abdullah Al-Othman, Tarek Hegazi, Mohammed Alsuhaimi, Sultan Alghamdi, Khaled Alenazi, Yazeed Alashban, Sami Alghamdi, Omar Quzi, Osama Jaafari, Saleh Alajlani, Abdulrahman Masmali, Yasser Hadi, Elbagir Manssor, Mustafa Mahmoud

**Affiliations:** 1 Department of Radiology and Medical Imaging, College of Applied Medical Sciences, Prince Sattam Bin Abdulaziz University, Alkharj 11942, Saudi Arabia; e.alkhybari@psau.edu.sa (E.A.); m.alshuhri@psau.edu.sa (M.A.); 2 Radiology Department, King Fahd Hospital of the University, Imam Abdulrahman Bin Faisal University, Dammam 34221, Saudi Arabia; ayothman@iau.edu.sa (A.A.-O.); tmhejazi@iau.edu.sa (T.H.); malsuhaimi@iau.edu.sa (M.A.); 3 Radiology and Nuclear Medicine Department, Security Force Hospital, Riyadh 11481, Saudi Arabia; sultan-661@hotmail.com; 4 Radiological Sciences Department, College of Applied Medical Sciences, King Saud University, Riyadh 11451, Saudi Arabia; kenazi@ksu.edu.sa (K.A.); yalashban@ksu.edu.sa (Y.A.); salghamdi1@ksu.edu.sa (S.A.); 5 Department of Radiology and Medical Imaging, King Fahad Central Hospital, Jazan Health Cluster, Jazan 82725, Saudi Arabia; oquzi@moh.gov.sa; 6 Radiology Department, Royal Commission Medical Centre, Industrial Yanbu, Yanbu 46451, Saudi Arabia; osipad2022@hotmail.com (O.J.); salajlani93@gmail.com (S.A.); masmali3006@gmail.com (A.M.); 7 Department of Medical Imaging and Intervention, King Abdullah Medical City, Makkah 57657, Saudi Arabia; hadi.y@kamc.med.sa; 8 Discipline of Medical Imaging and Radiation Therapy, University College Cork, T12 K8AF Cork, Ireland; 9 Department of Radiologic Technology, College of Applied Medical Sciences, University of Jeddah, Jeddah 23218, Saudi Arabia; ahali@uj.edu.sa; 10 Department of Radiological Sciences, College of Applied Medical Sciences, King Khalid University, Abha 62521, Saudi Arabia; malhassen@kku.edu.sa

**Keywords:** diagnostic reference levels, computed tomographic angiography, CT dose index volume, dose length product, multi-detector CT

## Abstract

Objectives: Diagnostic reference levels (DRLs) are crucial tools for optimizing radiation exposure during different radiological examinations. This study aimed to establish preliminary DRLs for commonly performed computed tomographic angiography (CTA) examinations in Saudi Arabia. Methods: Data for three types of CTA examinations (cerebral, pulmonary, and lower-extremity) were collected from six medical cities across Saudi Arabia. Data sets related to 723 CTAs with a mean patient weight of 75 kg were analysed in detail. The DRL values were determined based on the 75th, median, and 25th CT dose index volume (CTDI_vol_) and dose length product (DLP) values. Results: The established DRLs were 1221 mGy cm for cerebral CTAs, 475 mGy cm for pulmonary CTAs, and 1040 mGy cm for lower-extremity CTAs. These values were comparable to those reported in other studies. Conclusions: This study provides preliminary DRLs for three common CTA procedures in Saudi Arabia. The widespread implementation of a low kVp and a high level of image reconstruction (IR) presents an opportunity for further dose reduction. These findings can serve as a foundation for future nationwide DRL surveys and the optimization of CTA imaging protocols in Saudi Arabia.

## 1. Introduction

Imaging using multi-detector computed tomography (MDCT) scanners has rapidly developed over the last decade [1]. This technology started in 1998 with a 4-slice MDCT system and has continuously improved to the current 512-slice ultra-fast scanners [2]. The technological advancements that have been implemented in these CT systems, including fine detector elements in large-sized coverage detectors and iterative reconstruction software, has led to the acquisition of more data in a single rotation (a faster scan time) and an improved image quality (optimal spatial and temporal resolutions) [3,4]. This enhancement in image quality and post-processing tools in MDCT scanners has made CT angiographies (CTAs) effective imaging tools that can be used to assess the circulatory body system [5]. Clinically, CTA scans are recognized as essential procedures for demonstrating vascular anatomy and for confirming the presence of peripheral artery diseases in addition to routine clinical therapy [6]. This is because CTAs have an excellent diagnostic reliability with a specificity and sensitivity of at least 90% [7]. However, a literature review highlighted that some people who undergo CTA examinations may be exposed to a high radiation burden. The reported radiation dose quantities, including the dose length product (DLP) and volume CT dose index (CTDI_vol_), vary from 279 to 8374 mGy cm and from 2.3 to 23 mGy, respectively. Likewise, the effective dose delivered from CTA procedures varies from 1.7 mSv to 50 mSv [8]. Even though variations in radiation doses have been identified, dose optimization strategies for CTA examinations should be applied without deteriorating the image quality [7].

Radiation protection organizations have acknowledged that the optimization of radiation doses can be clinically managed by adopting the diagnostic reference level (DRL) concept. In 1996, the International Commission on Radiation Protection (ICRP) developed the concept of DRLs and subsequently introduced it into Europe [9,10]. DRLs can be assessed to monitor and then recognize abnormally high radiation doses by setting an upper threshold that standard radiation dose levels should not exceed when proper clinical practice is applied. DRLs can be established nationally in a country by gathering the data from different health facilities and reporting the upper threshold (75th percentile) for the most practical radiation doses. When a CT facility’s median radiation doses exceed the upper threshold of the national DRL (NDRL) standard, there is a need to optimize the radiation doses [9]. Therefore, it is important to establish an NDRL for CTAs to ensure that diagnostic findings are obtained during an exam while minimizing the unnecessary radiation burden.

The Saudi Food and Drug Authority (SFDA) is the central radiation organization responsible for reporting the NDRLs for diagnostic medical procedures in Saudi Arabia. Regarding CT scans, the SFDA reports the NDRLs for the CTDI_vol_ and DLP radiation doses related to adult CT procedures for the head, chest, abdomen, and pelvis [11]. However, the SFDA does not report the NDRLs for CTA examinations. Moreover, a literature search revealed that only three studies have established facility DRLs (FDRLs) for specific CTA examinations in Saudi Arabia. One study documented an FDRL value of 480 mGy cm for pulmonary CTAs [12]. Two additional FDRL studies reported DRLs for lower-extremity CTA examinations that ranged from 438 mGy cm to 3562 mGy cm [8,13]. In light of the limited availability of comprehensive DRL surveys for CTA examinations that have been established from a nationwide sample of hospitals across Saudi Arabia, this study endeavoured to address this gap by proposing and validating multiregional DRLs specifically tailored for routine CTA examinations. The paucity of existing DRLs derived from multicentre data within the Kingdom hinders the establishment of robust benchmarks for optimizing radiation dose practices in CTA procedures.

## 2. Materials and Methods

This research protocol received ethical approval from the Human Research Ethics Committee of the Imam Abdulrahman Bin Faisal University (IRP-PGS-2023-01-398). Ten medical facilities across the country were invited to participate in this retrospective study. Surveys were completed by six out of the ten hospitals approached.

A survey sheet was designed based on previous DRL works [14,15] to gather relevant CT scan data on adult patients undergoing common CTA examinations. Those examinations included cerebral, pulmonary, and lower-extremity CTAs. In accordance with ICRP Publication 135, a minimum of 30 CT examinations from each participating facility should be recorded to establish a DRL for a defined patient group undergoing a specific CT procedure [9].

A weight restriction criterion was not applied to the population sampling for this survey. However, the study population fell within the standard patient weight average (70 kg ± 15 kg) commonly used for establishing DRLs in CT examinations [16]. This study included adult patients (aged ≤ 18 years, encompassing both males and females) with a weight range of 50–95 kg.

The variables considered on the survey sheet included scanner characteristics, such as the name and model of the multi-detector CT (MDCT) system, and the CTA scanning parameters. The CT imaging protocol encompassed the following technical parameters: the tube current (mA), the tube potential (kVp), the rotation time, the slice thickness, and the pitch. Additionally, the level of iterative reconstruction (IR) employed was documented. Patient demographics, including their age, gender, and weight, were also collected. The DRL quantities, the volumetric CT dose index (CTDI_vol_), and the dose length product (DLP) were the radiation dose metrics used to report the DRL values, as recommended by the latest ICRP publication [9]. The dose quantities from the arterial phase were only included in the DRL dose survey to ensure data independency during the data collection process in order to standardize the reporting of DRLs. All the required variables were derived from the radiology information system (RIS) and the picture archiving and communication system (PACS) of each hospital. The data collection period encompassed examinations performed throughout 2023 and the first quarter of 2024.

To ensure an adequate image quality for the data used to propose the DRLs, only CTA examinations that were accepted and then reported by radiologists were reviewed. Furthermore, to guarantee an adequate image quality across participating CT facilities, annual quality assurance (QA) reports were obtained from the participating hospitals. This confirmed the implementation of a functional QA program for all the CT scanners used in this study.

The statistical analyses were conducted using IBM SPSS Statistics (version 24.0; IBM Corporation., Armonk, NY, USA). To establish facility-specific diagnostic reference levels (FDRLs), median values were calculated for both the CTDI_vol_ and the DLP across each CTA exam type within every CT facility. The 75th percentile for each measure was then rounded to determine the FDRL for each site. The results were communicated back to each participating CT facility. This process enabled the institutions to compare their FDRLs with established NDRLs. This feedback aimed to encourage the optimization of the CTA scan parameters for improved radiation dose management in these sites. Finally, the 75th, median, and 25th percentiles were calculated and recorded for each CT dose metric, and the DRL value was reported as the 75th percentile of both the CTDI_vol_ and the DLP.

## 3. Results

Six large hospitals from four regions participated in this study by completing and returning the survey data. The distribution across regions was as follows: two hospitals were in the central region, one was in the eastern region, two were in the western region, and one was in the southern region. A total of 725 CTA examinations were identified from these hospitals, performed on eight MDCT systems. To ensure the applicability of the established DRLs for CT examinations, two data sets were subsequently excluded due to involving extremely obese patients, leaving a final sample size of 723 CTAs for the analysis. The study sample included 246 cerebral CTA studies and 255 studies for pulmonary CTAs. Lower-extremity CTAs were carried out on 222 patients.

MDCT systems with 128 or more detector rows were the most frequently used (62% of scanners), while scanners with 64 slices were the second most frequently used systems in our study (38% of scanners). The MDCT scanner information included models from the following leading manufacturers: GE Light Speed (GE Healthcare, Milwaukee, USA; 25%), with 64 slices; GE Discovery CT750 HD (12.5), with 46 slices; GE Optima (12.5%), with 128 slices; Siemens Somatom Definition AS+ (Siemens Healthineers, Erlangen, Germany; 12.5%), with 128 slices; and Siemens Force (37.5%), with a dual source and 384 slices.

The study population included 53% male and 47% female participants. The mean age for all the participants was 59 years (SD = ±14 years). This male predominance (6% higher than females) aligned with the reported higher prevalence of cardiovascular disease (CVD) risk factors in males, suggesting a potentially high prevalence of vascular disease in the Saudi Arabian population [17]. The mean weight of the sampled population was 75 kg, which closely resembled the mean patient weight and standard deviation of 78 ± 15 kg reported in the published Saudi NDRL study for cardiac CTAs [14].

While 120 kVp constituted the standard tube voltage for most of the CTA examinations across the participating facilities, lower tube voltages of 100 or 80 kVp were routinely employed for lower-extremity CTA protocols compared to other anatomical regions. The tube current (mA) exhibited significant variation across the CTA protocols and MDCT systems, ranging from 63 mA to 957 mA. Newer MDCT systems, including dual-source scanners and the Somatom Definition Flash system, predominantly employed a pitch value of 1.2 or higher in 70% of the CTA examinations. Conversely, all the CTA examinations performed on 64-slice MDCT scanners utilized a pitch value of 1 or lower. The distribution of the IR levels utilized in the included CTA examinations demonstrated that a reduced IR level of 40% or less was employed in 15% of patients. An IR level of 50–60% was used in 10% of the patients, while the majority (75%) of CTAs utilized a level of 70% or higher. The scan length also exhibited variability across the cerebral, pulmonary, and lower-extremity CTA examinations, with means of 33 ± 10 cm, 28 ± 3, and 128 ± 19 cm, respectively (Table 1).

**Figure 1:**
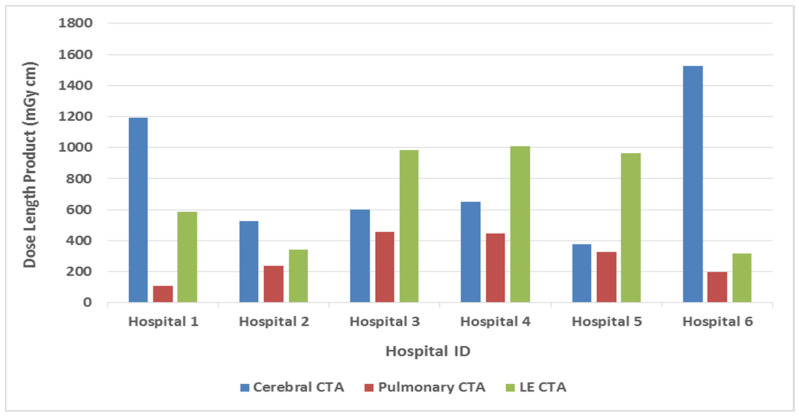
Distribution of facility diagnostic reference levels (FDRLs) per hospital.

Our results revealed significant variations in the DLP values across the participating hospitals for identical CTA examinations (Figure 1). This heterogeneity was attributable to the diverse scanning protocols employed by individual institutions (Table 2). Notably, for cerebral CTAs, Hospital 5 exhibited the highest DLP, exceeding the study’s median DLP by a factor of 2.4. Similarly, Hospital 4 demonstrated the highest DLP for lower-extremity CTAs, surpassing the median DLP by over 100%. Even though the pulmonary CTAs displayed the lowest overall DLP among the evaluated examinations, a substantial variation in the DLP (at least a 1.5-fold difference) persisted among hospitals. Table 3 presents the DRL quantities for the selected CTA examinations. Moreover, a comparison of these established DRLs with internationally reported values is presented in Table 4.

## 4. Discussion

The concept of DRLs was established in medical imaging as a key optimization tool for radiation dose management. This tool facilitates the practical monitoring and control of radiation exposures during radiological examinations without compromising the acquired image quality [9]. For the first time in Saudi Arabia, national DRL values for prevalent CTA examinations have been proposed, with the aim of achieving dose optimization in Saudi CT facilities (Table 3). The determined DRLs were 1221 mGy cm for cerebral CTAs, 475 mGy cm for pulmonary CTAs, and 1040 mGy cm for lower-extremity CTAs. It is noteworthy that these values are comparable to those reported in previously published studies on DRLs (Table 4).

For cerebral CTA examinations, our analysis showed substantially diverging DLP values in this study, with more than 20-fold differences (121–2832 mGy cm). A potential explanation for this divergence lies in the significant variation in the pitch values employed, as the pitch is inversely proportional to the dose quantity. In detail, Hospitals 1, 2, and 6 provided a compelling illustration of the influence of the pitch value on the DLP. These institutions utilized almost-identical scanner technology for cerebral CTA procedures. Notably, with minimal variation observed for the other scanning parameters, a significant difference in the median DLP emerged (Table 2). Hospitals 1 and 6 employed a pitch value of 0.7 and exhibited a 100% increase in the median DLP compared to Hospital 2, which utilized a pitch value of 1.2. The results were similar for lower-extremity CTA examinations. Hospital 6 employed a pitch value of 1.4 and achieved a significantly lower FDRL (316 mGy cm) compared to Hospital 4. In contrast, Hospital 4 utilized a lower pitch value (0.9) and exhibited a considerably higher FDRL (1036 mGy cm) (Figure 1). These findings underscore the critical role of pitch value selection in optimizing the radiation dose for state-of-the-art MDCT scanners. However, it is crucial to acknowledge the potential trade-offs associated with increasing the pitch value. Scanner-specific recommendations and a careful evaluation of benefits and drawbacks are essential when considering this approach.

Our analysis revealed that only 34% of the included cerebral CTA studies utilized a kVp setting of 100. Conversely, the majority of studies have employed kVp values of 120 and 140. However, the NDRLs for this CTA study were lower than the NDRLs reported by studies from different countries. Most DRLs for cerebral CTAs vary between 1224 and 4324 mGy cm (DLP), since surveys may not provide clear information regarding the scanning protocols employed and the number of sequences (phases) included in the reported data for cerebral CTAs (Table 4).

This study’s analysis showed that the resulting DLP value for pulmonary CTAs (475 mGy cm) was lower than those in most published NDRL studies, such as those reported in Kenya [12] (767 mGy cm), Ghana [24] (942 mGy cm), the USA [25] (557 mGy cm), and Qatar [26] (510 mGy cm). However, the corresponding CTDI_vol_ DRL value was higher in comparison with those of other studies (Table 4). This discrepancy might be attributable to the requirement for a concentrated radiation dose within the pulmonary region to counteract potential noise artifacts arising from the use of thinner slices (0.625 mm) in the acquisition of detailed reconstructed images. Moreover, the inter-facility comparison analysis of the FDRLs for pulmonary CTA examinations revealed a trend of comparable FDRLs across the participating hospitals. This observation was particularly pronounced among facilities that employed similar MDCT scanner technology. For example, Hospitals 3 and 4, which both utilized 64-slice scanners, exhibited near-identical FDRL values (Table 2). This finding can likely be attributed to the standardized scanning protocols implemented by these institutions for most CTA procedures.

In line with the limitations observed for common CTA examinations, existing DRL surveys for lower-extremity CTAs also appear to lack comprehensive data. Notably, only one NDRL study in Germany included DLP values for this specific type of CTA, with a reported value of 1000 mGy cm [18]. Our DLP value of 1040 mGy cm fell within this range, suggesting that a similar scanning protocol was used. However, our study identified a 25% increase in the median scan length compared to a previous German DRL survey. In our study, the scanning range commenced from the upper liver, whereas the German study initiated scanning at the pelvic area.

This study revealed a huge improvement in the MDCT systems used in Saudi Arabia; more than 62% of the included scanners were ≤128-detector elements, used dual head technology, and provided dose reduction features such as iterative reconstruction techniques, which maintain an optimal CTA image quality even with lower kVp settings. Despite these dose-saving technologies, the data confirmed a lack of awareness of using a low-kVp technique as a well-established strategy for reducing the radiation dose in CTA examinations. In those examinations in particular, an iodine contrast media was injected to highlight blood vessels. Lower-energy X-rays produced by lower kVp settings are better absorbed by iodine, resulting in a stronger signal for blood vessels compared to the surrounding tissues. Therefore, it is not surprising that significant variation in the DLP values was noted among the CTA examinations, as less than 34% of the included data points utilized a kVp of 100 or less.

Similarly, several studies have demonstrated that higher iterative reconstruction (IR) levels possess the greatest potential to reduce the radiation dose during CTA examinations [28,29]. This dose reduction is achieved through a significant reduction in the image noise, which may allow for the utilization of lower overall exposure levels while maintaining an acceptable image quality. Our findings corroborated these observations, as a substantial majority (75%) of the included CTA examinations employed IR levels of 70% or higher. However, it is concerning to note that one hospital implemented a fixed IR level of 30% for all the patients undergoing CTA examinations, regardless of their body habitus. This practice suggests a lack of individualization in scanning parameters, which might necessitate a thorough revision to optimize the image quality and radiation dose.

There are several factors that contributed to our study, demonstrating comparable or even lower DRL values compared to the majority of published studies. First, our data originated from different hospitals nationwide that used advanced and state-of-the-art MDCT systems with at least 128 slices and dual-source technology, while other studies involved data from CTA scans acquired using older scanner models such as 64-slice scanners. Second, only the radiation dose associated with the arterial phase was included in the process of determining the DRL values in this study, whereas other studies have incorporated data from multiple scan phases (two or three). However, our analysis revealed significant variations in the FDRLs across the participating hospitals. This heterogeneity can be primarily attributed to discrepancies in the acquisition protocols, particularly the scan length, kVp, pitch, and level of IR (Table 2). These findings highlight the critical need for standardization in the scanning parameters of CTA examinations. Furthermore, promoting the adoption of established dose-saving techniques, such as the utilization of low-kVp and high-IR methods, is crucial. Educational initiatives and training programs should be implemented to equip technologists and radiologists with the knowledge and skills necessary to confidently employ these methods. Such efforts will optimize the image quality for making accurate diagnoses while minimizing the radiation exposure of patients.

This study has limitations that warrant consideration. Firstly, the scope of the study was confined to three specific types of CTA examinations. Future investigations should aim to encompass a broader range of CTA scan types, including those for the carotid and thoracic–abdominal regions. This will contribute to the establishment of a more comprehensive set of DRLs that can be applied across various CTA procedures. Secondly, the DRL values in this study were determined based only on the CTDI_vol_ and DLP values. Notably, a size-specific dose estimate (SSDE), which represents the actual dose received by patients of various body habitus, was not proposed [30]. Despite these limitations, these findings can serve as a valuable foundation for optimizing the scan protocols used by the participating facilities. We acknowledge the need for further research to establish a more comprehensive set of DRLs that are applicable to a broader spectrum of CTA examinations across Saudi Arabia. This future endeavour will likely be undertaken by the SFDA in a nationwide DRL survey.

## 5. Conclusions

In conclusion, this study investigated the DRLs for three frequently performed CTA procedures in Saudi Arabia: cerebral, pulmonary, and lower-extremity CTAs. The established DRLs were 1221 mGy cm for cerebral CTAs, 475 mGy cm for pulmonary CTAs, and 1040 mGy cm for lower-extremity CTAs. These values are comparable to the DRLs reported in other published studies. An analysis of the facility DRLs across the participating hospitals revealed significant inter-facility variations. This heterogeneity appears to be strongly linked to discrepancies in the employed scanning parameters, particularly the pitch value and kVp. Our findings suggest that the widespread adoption of dose-saving techniques, such as a low kVp and high IR levels, has the potential to further reduce radiation exposure during CTA imaging in Saudi Arabia.

## Figures and Tables

**Table 1:** Descriptive statistics of patient and scanning parameters used in CTA imaging at participating CT facilities.

Patient/Scan Parameters (Mean ± SD)	CTA Examination		
	Cerebral CTA	Pulmonary CTA	Lower-Extremity CTA
Gender (%)	Male: 57% Female: 43%	Male: 44% Female: 56%	Male: 69% Female: 31%
Patient Age	50 ±18	49 ± 19	46 ± 18
Weight	70 ± 16	76 ± 22	74 ± 18
Tube Voltage (kVp)	111 ± 17	114 ± 16	103 ± 16
Tube Loading (mA)	636 ± 348	395 ± 197	263 ± 144
Pitch	1 ± 0.3	1 ± 0.4	1 ± 0.3
Slice Thickness (mm)	0.8 ± 0.6	1.2 ± 1	0.9 ± 0.7
Scan Length (cm)	33 ± 10	28 ± 3	128 ± 19
Level of Iterative Reconstruction	60% ± 22%	52% ± 23%	65% ± 20%
Volumetric CT Dose Index (CTDI_vol_)	30 ± 14	11 ± 6	7 ± 5
Dose Length Product (DLP)	2213 ± 486	335 ± 187	780 ± 362

**Table 2:** Median value of imaging factors used in routine CTA examinations according to CT scanner.

Hospital	CTA Examination	Technical Factors (Median)								
		Scanner Model	Detector Row/Slice	kVp	mA	Pitch	Slice Thickness (mm)	Scan Length (cm)	Level of IR (%)	DLP
Hospital 1	Cerebral	Somatom Force	384	100	850	0.7	0.75	35.3	-	1192
	Pulmonary			90	355	1.9	0.75	33	-	107
	Lower-Extremity			120	421	1.5	0.75	126	-	588
Hospital 2	Cerebral	Somatom Force GE Optima CT 660CT2	384 64	100	183	1.2	0.625	38.4	30	527
	Pulmonary			120	130	0.55	0.625	36	30	237
	Lower-Extremity			80	138	0.6	0.625	83.4	30	351
Hospital 3	Cerebral	GE Light Speed	64	120	590	0.9	0.625	43.5	70	619
	Pulmonary			120	525	0.9	0.625	30	70	459
	Lower-Extremity			100	245	1	0.625	124	70	990
Hospital 4	Cerebral	GE Light Speed	64	120	514	0.9	0.625	39	70	627
	Pulmonary			120	519	0.9	0.625	28	70	442
	Lower-Extremity			100	280	0.9	0.625	123	70	1036
Hospital 5	Cerebral	GE Discovery CT750 HD	128	120	600	0.9	0.625	33	80	640
	Pulmonary			120	449	1.4	0.625	32.8	80	327
	Lower-Extremity			120	406	0.9	0.625	145	80	963
Hospital 6	Cerebral	Somatom Definition Flash	-	100	860	0.7	0.625	39.5	55	1525
	Pulmonary			100	478	1.2	0.625	33.4	80	197
	Lower-Extremity			100	121	1.4	0.625	133	80	316

**Table 3:** DRLs for common CTA examinations in Saudi Arabia.

Scan Type	CTDI_vol_ (mGy)			DLP (mGy cm)		
	75th	Median	25th	75th	Median	25th
Cerebral CTA	40	30	16	1221	643	440
Pulmonary CTA	15	10	5	475	328	155
Lower-Extremity CTA	9	7	3	1040	837	440

**Table 4:** Comparison of proposed DRL quantities (CTDI_vol_ and DLP) for common CTA examinations with international DRL values.

Scan Type	CTDI_vol_ (mGy)		DLP (mGy cm)	
	NDRLs	DRLs	NDRLs	DRLs
Cerebral CTA	40	20 [18], 65 [19], 74 [20], 43 [21], 50 [22]	1221	600 [18], 1134 [19], 1224 [20], 1850 [21], 4324 [22]
Pulmonary CTA	15	15 [19], 13 [22], 8 [23], 9 [10], 14 [24], 18 [25], 8 [26], 13 [27]	475	450 [19], 767 [22], 310 [23], 310 [10], 942 [14], 557 [25], 510 [26], 430 [27]
Lower-Extremity CTA	9	8 [18]	1040	1000 [18]

## Data Availability

A full data set is available upon request to the corresponding author.
